# A Hybrid Transformer-Convolutional Neural Network for Segmentation of Intracerebral Hemorrhage and Perihematomal Edema on Non-Contrast Head Computed Tomography (CT) with Uncertainty Quantification to Improve Confidence

**DOI:** 10.3390/bioengineering11121274

**Published:** 2024-12-15

**Authors:** Anh T. Tran, Dmitriy Desser, Tal Zeevi, Gaby Abou Karam, Fiona Dierksen, Andrea Dell’Orco, Helge Kniep, Uta Hanning, Jens Fiehler, Julia Zietz, Pina C. Sanelli, Ajay Malhotra, James S. Duncan, Sanjay Aneja, Guido J. Falcone, Adnan I. Qureshi, Kevin N. Sheth, Jawed Nawabi, Seyedmehdi Payabvash

**Affiliations:** 1 Department of Radiology and Biomedical Imaging, Yale School of Medicine, New Haven, CT 06510, USA; 2 Department of Neuroradiology, Charité—Universitätsmedizin Berlin, Humboldt-Universität Zu Berlin, Freie Universität Berlin, Berlin Institute of Health, 10117 Berlin, Germany; 3 Department of Neuroradiology, University Medical Center Hamburg-Eppendorf, 20246 Hamburg, Germany; 4 Department of Radiology, Zucker School of Medicine at Hofstra/Northwell, Manhasset, NY 11030, USA; 5 Department of Radiation Oncology, Yale School of Medicine, New Haven, CT 06510, USA; 6 Department of Neurology, Yale School of Medicine, New Haven, CT 06510, USA; 7 Zeenat Qureshi Stroke Institute and Department of Neurology, University of Missouri, Columbia, MO 65211, USA

**Keywords:** intracerebral hemorrhage, perihematomal edema, segmentation

## Abstract

Intracerebral hemorrhage (ICH) and perihematomal edema (PHE) are key imaging markers of primary and secondary brain injury in hemorrhagic stroke. Accurate segmentation and quantification of ICH and PHE can help with prognostication and guide treatment planning. In this study, we combined Swin-Unet Transformers with nnU-NETv2 convolutional network for segmentation of ICH and PHE on non-contrast head CTs. We also applied test-time data augmentations to assess individual-level prediction uncertainty, ensuring high confidence in prediction. The model was trained on 1782 CT scans from a multicentric trial and tested in two independent datasets from Yale (n = 396) and University of Berlin Charité Hospital and University Medical Center Hamburg-Eppendorf (n = 943). Model performance was evaluated with the Dice coefficient and Volume Similarity (VS). Our dual Swin-nnUNET model achieved a median (95% confidence interval) Dice = 0.93 (0.90–0.95) and VS = 0.97 (0.95–0.98) for ICH, and Dice = 0.70 (0.64–0.75) and VS = 0.87 (0.80–0.93) for PHE segmentation in the Yale cohort. Dice = 0.86 (0.80–0.90) and VS = 0.91 (0.85–0.95) for ICH and Dice = 0.65 (0.56–0.70) and VS = 0.86 (0.77–0.93) for PHE segmentation in the Berlin/Hamburg-Eppendorf cohort. Prediction uncertainty was associated with lower segmentation accuracy, smaller ICH/PHE volumes, and infratentorial location. Our results highlight the benefits of a dual transformer-convolutional neural network architecture for ICH/PHE segmentation and test-time augmentation for uncertainty quantification.

## 1. Introduction

Spontaneous intracerebral hemorrhage (ICH) is the most devastating form of stroke [1]. While ICH accounts for 10–15% of all acute strokes [2], the global burden of hemorrhagic stroke, measured by disability-adjusted life years lost, is greater than that of ischemic stroke [3]. The estimated annual cost of care and productivity losses from ICH in the United States is approximately 12.7 billion dollars [4]. Prognostic tools in acute ICH can help with establishing care goals, characterizing and selecting patients for interventions, and facilitating informed discussions with patients and their families regarding prognosis. The ICH and perihematomal edema (PHE) volumes have emerged as two main neuroimaging predictors of outcome in ICH and, thus, important components in prognostic models [5].

The damage to brain parenchyma caused by spontaneous ICH involves both primary injury, resulting from the mechanical impact of hemorrhage and its expansion, and secondary injury, arising from inflammatory cascades activated in response to the neurotoxic products of blood breakdown and leakage from the blood–brain barrier [6]. For any given location in the brain, ICH volume is the most potent imaging predictor of 30-day mortality rates [7]. PHE is the radiological marker of secondary brain injury and is increasingly used as a surrogate metric to assess the potential efficacy of treatments targeting secondary brain injury in ICH [8]. A recent meta-analysis of 20 studies revealed that the PHE volume at baseline and its growth over the first 24 h after ICH onset will impact outcomes [8]. Accurate volumetric measurements of ICH and PHE in hemorrhagic stroke have prognostic implications and potential roles in guiding treatment and clinical trial enrollment. However, manual quantification of ICH and PHE volumes from brain scans is time-consuming and inefficient. Automated segmentation tools can facilitate timely quantification of ICH and PHE volumes from brain scans in the acute stroke setting.

Non-contrast computed tomography (CT) is the most widely accessible and the primary imaging modality for patients with spontaneous ICH. Traditional approaches that rely on threshold-based segmentation and edge detection algorithms have limitations for segmenting ICH and PHE lesions on CTs. The inherent similarity in CT Hounsfield Unit (HU) density between PHE, cerebrospinal fluid, and other pathologies hinders accurate differentiation. Indeed, manual PHE segmentation methods heavily depend on radiologists’ expertise in distinguishing PHE from pre-existing brain injuries. Artificial intelligence algorithms, specifically tailored for accurate segmentation of ICH and PHE from head CT scans, offer the potential for expeditated and reliable quantification of these biomarkers.

While many groups have reported various deep learning models for ICH segmentation in non-contrast head CTs, there is a significant paucity of models that simultaneously segment ICH and PHE. In this study, we aimed to develop and externally validate a novel hybrid model combining convolutional neural networks (CNNs) and transformers for the segmentation of ICH and PHE on non-contrast head CTs, with the inclusion of prediction uncertainty measures. The hybrid transformer–CNN architecture can leverage CNNs’ strength in extracting local features and the transformers’ ability to capture global context and long-range dependencies. Additionally, incorporating prediction uncertainty measures enables users to identify scans where the model had low confidence in segmentation results, warranting further review. Finally, to facilitate broader adoption of our model, we packaged and publicly shared the segmentation pipeline as a Graphical User Interface (GUI) application.

In this study, we created an end-to-end fully automated pipeline that can pre-process and simultaneously segment ICH and PHE from non-contrast head CTs with high accuracy. We combined a vision transformer and a U-Net-based model with multiple output resolutions and loss functions and applied test-time augmentation to detect uncertainty in individual-level segmentation results. Finally, the segmentation model is embedded in a Graphical User Interface (GUI) application and publicly shared.

## 2. Materials and Methods

### 2.1. Study Population

**Figure 1:**
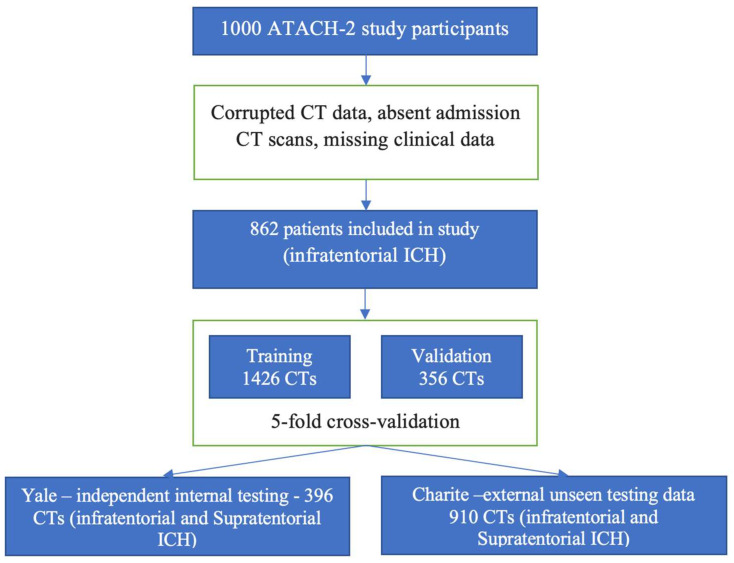
Diagram of datasets used for training, validation, and testing of our model.

Figure 1 summarizes the three datasets used for training/cross-validation, internal independent test, and external unseen test in this study. The demographic, clinical, and imaging characteristics of each dataset are summarized in Supplementary Table S1. All head CT scans were obtained within the first 36 h after ICH onset.

For the training/cross-validation dataset, we used the head CTs from the Antihypertensive Treatment of Acute Cerebral Hemorrhage II (ATACH-2) trial [9]. ATACH-2 was a multicenter randomized trial that enrolled patients with acute supratentorial ICH within 4.5 h of onset, baseline hematoma < 60 mL, and systolic blood pressure elevation (>180 mm Hg). After excluding CT scans with artifacts or procedures affecting the ICH or PHE lesions, we included 1782 CTs from 893 patients (890 with both baseline and follow up, and 2 with only baseline CT) [10,11,12].

For independent testing of the model, we included consecutive patients with acute spontaneous ICH between 1 January 2018 and 31 December 2021 from the Yale Longitudinal Study of Acute Brain Injury [13]. This single-center database has prospectively collected information on patients with acute brain injury including ICH. From this dataset, we included 396 CT scans from 202 patients (194 with baseline and 24 h follow-up CT scans, and 8 with only baseline CT).

For external validation of models in unseen dataset, we used a multi-centric cohort of consecutive patients with acute spontaneous ICH who presented to the Charité University Hospital in Berlin and the University Medical Center Hamburg-Eppendorf between 1 January 2015 and 31 December 2019, as described previously [14].

### 2.2. Manual Segmentation of Lesion for the Ground Truth

The ICH and PHE lesions were manually segmented by trained radiology residents and research associates under the supervision of board-certified neuroradiologists at Yale (SP) and Charité University Hospital in Berlin (JN). Each reviewer was initially trained on a subset of at least 100 head CTs. Supervising expert neuroradiologists (SP and JN) thoroughly reviewed all ICH and PHE segmentations and verified their accuracy, providing continuous feedback to maintain and improve the segmentation process. The research team at Yale utilized 3D-slicer and MRIcron [10,11,12]; and the research team at Charité University Hospital used ITK-SNAP freeware [14,15,16] for the manual segmentation of lesions on axial slices. A subset of scans at each center was segmented twice more: once by the original reviewer and once by an independent second reviewer. Thus, both research teams determined intra- and inter-rater reliability of segmentations using intra-class correlation (ICC), as reported previously [10,11,12,14,15,16].

Both research teams applied HU intensities to guide segmentation of ICH and PHE as reported previously [10,11,12,14,15,16,17]. Separation of PHE from pre-existing leukoaraiosis was based on neuroanatomical distribution of edema, which typically surrounds hematoma versus relatively symmetric pattern of leukoaraiosis [10,16]. Of note, separating ICH from IVH on head CT is particularly challenging due to overlapping HUs of acute blood in the parenchyma and ventricular system, the closed proximity of hematoma in parenchymal tissue with IVH, and deformation of the ventricular system from hematoma mass effect or developing hydrocephalus. Considering these constraints, both research teams separated ICH from IVH by following the contours of cerebral ventricles, paying attention to potential ventricular effacement or dilation deformities.

### 2.3. Preprocessing of Head CTs

We preprocessed all non-contrast head CTs by removing the osseous skull, adjusting the images to CT brain window level, cropping, and resampling:

- Skull stripping: Based on air, fatty tissue, and bone densities on non-contrast head CT, we removed voxels with an intensity <0 and >200 HU to facilitate skull stripping. After removing much of the skull using these HU thresholds, we applied morphology-based methods with image dilation and erosion operations to remove remaining soft tissue structures.
- Brain windowing: Radiologists usually apply specific window width and level to accentuate the contrast between target tissues. The optimal setting for visual inspection of brain tissue on non-contrast CTs is a window level = 40 and width = 80 that we applied in our pre-processing.
- Cropping and resampling: The 3D images were automatically cropped using a bounding box around the foreground object (brain tissue), reducing the image size while preserving objects. Then, we resampled all images and segmentation masks to the mean spacing of training set. This will mitigate the heterogeneity in slice thickness and voxel dimensions across different head CTs and provide consistent voxel spacing for the model.

### 2.4. Dual Model Structure

**Figure 2:**
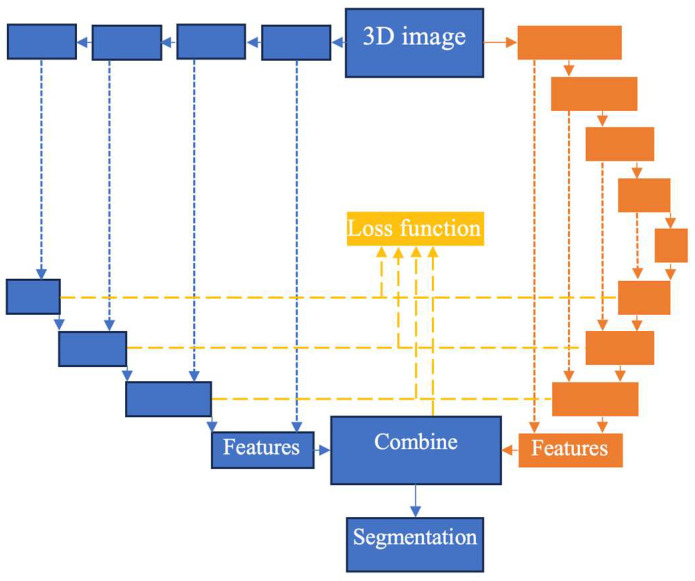
A combination of two models for segmentation and extracting multiple outputs for loss function. Blue color represents SwinUNETR, orange color represents nnU-NETv2, and yellow represents the integration of loss function.

Each deep learning model has its strengths and weaknesses, as different CNN architectures process input data in different ways. Combining these models can provide a complementary and more comprehensive understanding of features, improving segmentation performance. Among Transformers, the SwinUNETR has the advantage of using Shifted windows for computing self-attention [18]. The Swin transformer encoder extracts features at five different resolutions and is connected to a decoder while traditional UNET uses convolution, max-pooling layers from the original images. Hybrid models can combine local feature extraction by traditional convolutional networks with global context features captured by transformers [19]. nnU-NETv2 is designed to automatically configure itself to the dataset (e.g., preprocessing, augmentation, model architecture tuning), reducing manual overhead. Therefore, we integrate SwinUNETR into the nnUNET framework to provide better delineation of boundaries, improved handling of small structures, and more consistent predictions across complex datasets. A 3D input image is randomly split into multiple batches. Each batch was segmented using two different models. In each model, there are six segmentation results at different resolutions. The loss function minimizes the difference between six results and ground truth. The last layer is the output from batch segmentation results (Figure 2). Then, all batch segmentation results are combined into the final segmentation.

Swin-Unet transformers are computationally heavy due to their self-attention operations, especially for high-resolution inputs. Combining them with nnU-NETv2 could result in a significantly larger model. Training and inference may demand high-end GPUs and more time, making the approach less feasible for resource-constrained environments. Seamlessly integrating the transformer-based Swin-Unet components with nnU-NETv2′s convolutional framework may require significant architectural redesign. A common way to solve the problem is by resizing images to smaller dimensions. However, this approach may lose image details that are important for segmentation of small objects. We applied sliding window inference, where images are split into overlapping sliding windows that serve as inputs for training, and their segmentation results are combined afterward. This approach allows for handling large images by processing them in smaller patches, which is particularly beneficial in memory-limited environments.

Algorithm 1 shows the details of training and validating process for our model.
**Algorithm 1:** Train and validate our CNN model**Input:**  I is the set of training images x after pre-processing and ground truth images y  V is the set of validation images x after pre-processing and ground truth images y  epoch is the number of running all training data. **Output:**  $W_{seg}\;$$\;\mathrm{is}\;\mathrm{the}\;\mathrm{weight}\;\mathrm{of}\;\mathrm{combination}\;\mathrm{CNN}\;\mathrm{segmentation}\;\mathrm{model}\;{f(s,\;W}_{fseg})\;and\;{g(s,\;W}_{gseg})$ for a sliding window s **Let** $S\left(x\right)=\left(s_{0},s_{1},\ldots ,\;s_{k}\right)$ be a set of k sliding windows for image x **Let** $G\left(x\right)=(g_{0},g_{1},\ldots ,\;g_{k})$ be a set ground truth of k sliding windows for image x **For** epoch:1,…,number of epoch:  For x∈I:   $\mathrm{For}\;\mathrm{each}\;(s_{i},\;g_{i})$$\;\mathrm{where}\;s_{i}\in S\left(x\right)\;and\;g_{i}\in G\left(x\right)$ do:    $\;{Seg(seg}_{0},{seg}_{1},\ldots {,seg}_{m})\leftarrow f(s_{i},\;W_{fseg})\cup \;g(s_{i},\;W_{gseg})$   $\mathrm{Let}\;R\left(g_{i}\right)=\left(r_{0},r_{1},\ldots ,\;r_{m}\right)$$\;\mathrm{be}\;\mathrm{the}\;\mathrm{multiple}\;\mathrm{resolution}\;\mathrm{ground}\;\mathrm{truth}\;\mathrm{of}\;\mathrm{sliding}\;\mathrm{window}\;g_{i}$   Minimize loss function L(Seg, R)   $\mathrm{Update}\;\mathrm{parameter}\;\mathrm{and}\;\mathrm{weight}\;W_{seg}$  For x∈V do:   $\mathrm{For}\;\mathrm{each}\;(s_{i},\;g_{i})$$\;\mathrm{where}\;s_{i}\in S\left(x\right)\;and\;g_{i}\in G\left(x\right)$ do:     Validate ($S\left(x\right)$, $G\left(x\right)$*)*      ${Seg(seg}_{0},{seg}_{1},\ldots {,seg}_{m})\leftarrow f(s_{i},\;W_{fseg})\;\cup \;g(s_{i},\;W_{gseg})$   ${metric\leftarrow (seg}_{0},g_{i}$) **Check and save weight** $W_{seg}$


### 2.5. Multiple Loss Function for Multiple Output Segmentation

We used the cross-entropy loss [20], a popular loss function for (pixel-wise) classification combined with the Dice coefficient [21], which measures the volumetric overlap between segmentation results and ground truth (details are described in Supplementary Materials). Each model has a dual architecture, comprising an encoder and a decoder. The model generates an output for every decoder layer and incorporates them as inputs into the combination of two loss functions. This design is essential for the backpropagation algorithm, which is used to update the model’s weights and biases during training effectively. The architecture of combined model for segmentation and extracting outputs for loss function is shown in Figure 2.

### 2.6. Uncertainty Detection

Different methods have been proposed for detection of prediction uncertainty in deep learning models, including Bayesian Neural Networks [22] and test-time augmentation [23,24]. Because the object for segmentation is small, we applied test-time data augmentation and defined an Uncertainty Score when running n times segmentation. The value of Uncertainty Score∈[0,1]

(1) $${Uncertainty\;Score}_{x}=\frac{\sum_{i=1}^{n}\frac{\left|V_{i}-V_{0}\right|}{\left|V_{i}+V_{0}\right|}}{n}$$

where $V_{0}$ is the volume of original segmentation, $V_{i}$ is the volume of x segmentation at the ith time, $V_{i}+V_{0}>0$.

Notably, the ICH and PHE segmentation results are averaged in each subject to calculate the Uncertainty Score.

### 2.7. Statistics and Evaluation of Segmentation Performance

To evaluate the performance of the segmentation models, we used three metrics: Dice coefficient, Hausdorff distance (HD), and Volume Similarity (VS), detailed in the Supplementary Material. Briefly, the Dice coefficient measures the area overlap between segmentation results and ground truth. HD assesses the maximum surface distance [25], specifically the maximum distance from a point in one set to the nearest point in the other set. VS quantifies the similarity between the ground truth and segmented volumes, ranging from 0 to 1, where 1 indicates perfect volume similarity and values closer to 0 indicate poorer similarity.

### 2.8. Training and Cross-Validation

For training and hyperparameter optimization, we applied a 5-fold cross-validation scheme on n = 1782 CTs from the ATACH-2 trial. Using optimized hyperparameters, we trained the final model on a whole training/cross-validation dataset. The trained model was then tested on n = 396 CTs from the independent Yale test set and n = 943 CT scans from the external unseen test set at Charité University Hospital.

**Figure 3:**
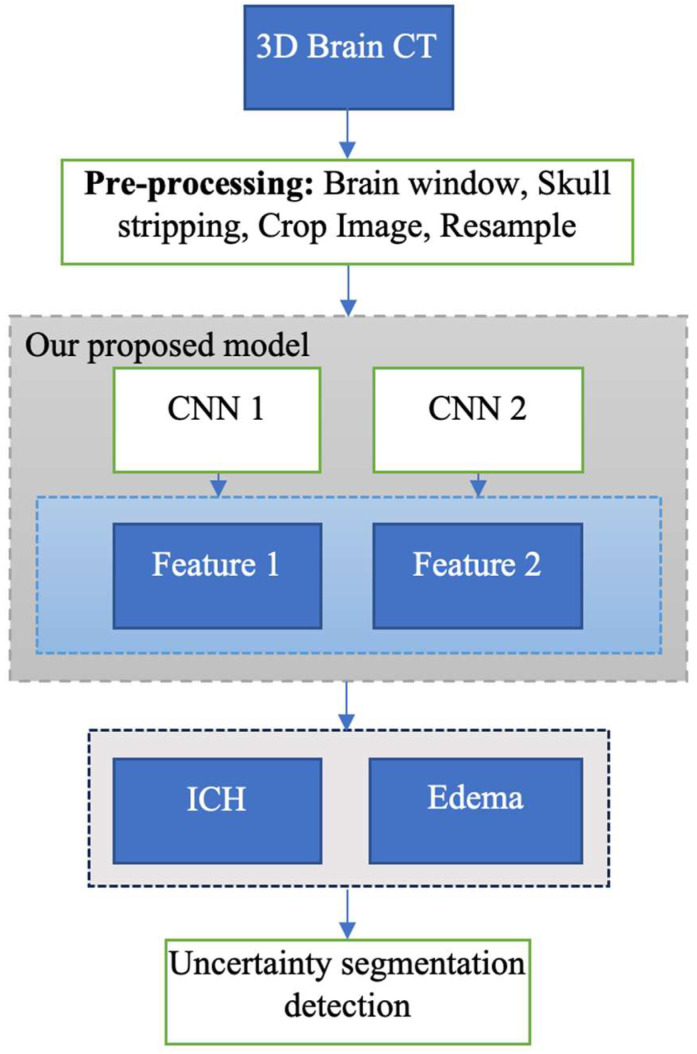
The workflow of end-to-end automated pipeline for segmentation of intracerebral hemorrhage (ICH) and perihematomal edema (PHE) on non-contrast head CT scans.

In the preprocessing step, each 3D brain CT scan is skull-stripped, adjusted to CT brain window level, cropped, and resampled to 3.75 mm × 0.46 mm × 0.46 mm spacing, based on average from the ATACH-2 trial dataset. After segmentation, the final masks are reverted to the original head CT scan dimension for comparison against the ground truth. The final pipeline is shown in Figure 3. We used augmentation, weight_decay = 3 × 10^−5^, initial_lr = 1 × 10^−2^, num_epochs = 100, PolyLRScheduler, optimizer = SGD, and patch size = (32,256,256).

**Figure 4:**
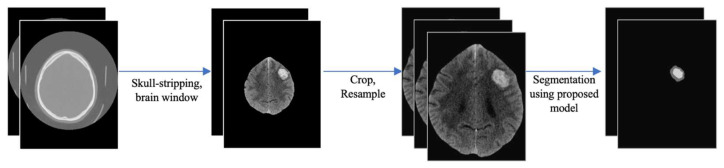
An example of ICH and PHE segmentation of a CT following the pipeline.

We also implemented and compared our model with state-of-art segmentation methods including SegResNet, SwinUNETR, and nnUNET following the open source for ICH segmentation Deepbleed [26]. The pipelines for these models had five main steps: extracting brain windows, skull stripping, registration, segmentation, and reverting to the original size (Figure 4). The output has two labels: ICH and PHE. The template for registration has spacing of (1.5, 1.5, 1.5) and has size of (128,128,128). We used similar augmentation strategies, weight_decay = 3 × 10^−5^, initial_lr = 1 × 10^−4^, num_epochs = 100, CosineAnnealingLR, optimizer = Adam, loss_function = DiceFocalLoss, and patch size = (64,64,64).

All experiments at Yale were carried out by a computing device with an AMD Ryzen 397SWX 32 Cores 2200H CPU (52 GB memory) (Advanced Micro Devices, Inc., Santa Clara, CA, USA) and 4 GPUs (NVIDIA Quadro RTX 6000, NVIDIA, Santa Clara, CA, USA) with 4 × 24 GB memory. All work was conducted using the Ubuntu 20 operating system and Python 3.8. External unseen testing, in University of Berlin Charité Hospital, was performed on a PC with an NVIDIA GeForce RTX 3090 (NVIDIA, Santa Clara, CA, USA).

### 2.9. Graphical User Interface (GUI) Application

**Figure 5:**
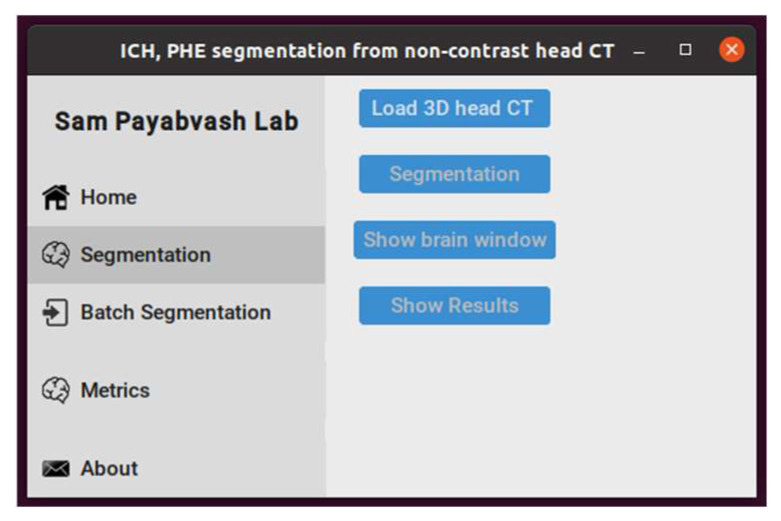
The interface of GUI application for hematoma and edema segmentation from non-contrast head CT.

To facilitate model sharing and inter-institutional collaboration, we developed a GUI application incorporating the final segmentation model (Figure 5). We applied a three-tier application architecture to build the GUI, including the presentation, business, and model tiers. We used the CustomTkinter Python UI library, based on Tkinter, in the presentation tier, which provides fully customizable widgets (Figure 5). We used the PyInstaller libraries to deploy the application. The advantage of our GUI application is providing a standalone tool, where users can run it locally instead of uploading to a web server preserving data security.

**Figure 6:**
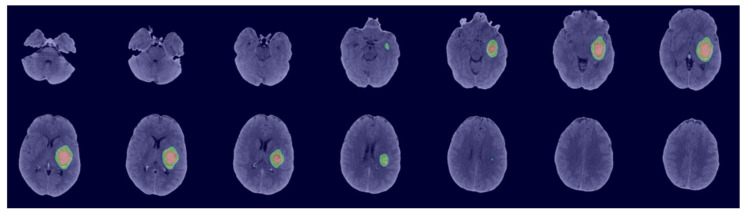
An example of ICH and PHE segmentations from non-contrast head CT using our GUI application.

Figure 6 shows an example of the GUI application output. Notably, our GUI application includes all necessary libraries to integrate with the local Python environment and run on any operating system, resulting in a final application file size of 9 GB. We publicly shared the GUI application on the NeuroImaging Tools and Resources Collaboratory (NITRC) data repository website: https://www.nitrc.org/projects/ichphesegment (accessed on 10 December 2024).

## 3. Results

### 3.1. Model Performance

Table 1 lists the mean ± standard deviation and median (interquartile) Dice coefficients, HD (mm), and VS from validation folds of the 5-fold cross-validation in the ATACH-2 dataset (n = 1782 CT scans) as well as testing the final model in the internal test cohort from Yale (n = 396) and external unseen test cohort from the Charité University Hospital in Berlin and the University Medical Center Hamburg-Eppendorf (n = 943). Compared to SegResNet and SwinUNETR models, our dual-architecture model integrating SwinUNETR into nnU-NETv2 with multiple output resolutions for multiple loss validation achieved higher Dice and VS. In the internal test cohort, the Swin-nnUNET segmentations of ICH and PHE had significantly higher Dice and VS (all *p* < 0.001) compared to SwinUNETR using a paired-sample *t*-test comparison. There was also a trend toward lower HD in ICH (*p* = 0.150) and PHE (*p* = 0.310) segmentation by dual Swin-nnUNET compared to SwinUNETR. In the external test cohort, the dual Swin-nnUNET segmentations of ICH and PHE had significantly higher Dice and VS, and lower HD compared to SwinUNETR (all *p* < 0.001). Comparing Swin-nnUNET versus nnU-NET, the dual Swin-nnUNET model achieved higher Dice (*p* = 0.030) and VS (*p* < 0.001) and lower HD (*p* = 0.001) for PHE segmentation in the internal test cohort; there was also a lower HD for ICH and PHE segmentation in the external unseen test cohort (both *p* < 0.001).

### 3.2. Detection of Uncertainty in Segmentation

The segmentation performance of our proposed dual Swin-nnUNET model, excluding uncertain segmentations, is summarized in Table 1. Using test-time augmentation and uncertainty scoring, we identified segmentation uncertainty in 7 out of 396 CT scans in the internal testing cohort and 24 out of 943 CT scans in the external unseen testing cohort. In both test cohorts, subjects with uncertain segmentations had lower Dice and VS and higher HD compared to those with certain segmentations (see Supplementary Table S3). In the internal test cohort, uncertain segmentations had smaller ICH (5.5 ± 9.2 mL versus 16.0 ± 16.9 mL, *p* = 0.103) and PHE (4.9 ± 7.6 mL versus 16.4 ± 14.6 mL, *p* = 0.040) volumes than those without uncertainty. The rate of infratentorial hemorrhage was higher in uncertain segmentations (4 out of 7) than those without uncertainty (21 out of 195, *p* < 0.001). In the external unseen test cohort, uncertain segmentations also had smaller ICH (3.8 ± 8.5 mL versus 36.6 ± 36.4 mL, *p* < 0.001) and PHE (3.8 ± 4.7 mL versus 33.1 ± 34.1 mL, *p* < 0.001) volumes than those without uncertainty. The rate of infratentorial hemorrhage was higher in uncertain segmentations (10 out of 24) compared to those without uncertainty (142 out of 919, *p* < 0.001).

### 3.3. Factors Affecting Segmentation Accuracy

We examined the relationship between ICH, PHE, and IVH volumes, ICH location (supra- versus infratentorial and lobar versus deep), scan parameters, time from onset to scan, and patients’ age with segmentation accuracy.

Given that ICH and PHE volumes were smaller in the ATACH-2 training/cross-validation cohort compared to the external validation (Supplementary Table S1), we examined the relationship between the ground truth volumes and HD in both internal and external test cohorts. We used the HD metric since the Dice coefficient and VS have inherent collinearity with lesion volumes. In the Yale internal testing cohort, there were no significant associations between ICH (r = −0.05, *p* = 0.311) and PHE (r = 0.02, *p* = 0.688) volumes with segmentation HD. In the Charité Hospital external testing cohort, there was also no significant association between ICH volume and HD (r = 0.01, *p* = 0.766), but larger PHE volumes were associated with lower HD (r = −0.05, *p* < 0.001).

Since separation of ICH from IVH is intrinsically challenging, we also analyzed the relationship between the IVH volume and segmentation accuracy. There was no significant correlation between IVH volume with ICH (Pearson r = −0.12, *p* = 0.09) or PHE (r = 0.17, *p* = 0.82) segmentation Dice in the Yale dataset, but we found a negative correlation between IVH volume with ICH (r = −0.29, *p* < 0.001) and PHE (r = −0.20, *p* < 0.001) segmentation Dice in the Charité Hospital external testing cohort.

As infratentorial ICH was almost absent in the training cohort, we examined the effects of supra- versus infratentorial location on segmentation accuracy. Supplementary Table S2 provides details of models’ performance in supra- versus infratentorial hemorrhage for both internal and external unseen test cohorts. In the internal test cohort, infratentorial ICH segmentation had lower mean Dice and VS, and higher HD (all *p* < 0.001), compared to supratentorial ICH. Similarly, infratentorial PHE segmentation had lower mean Dice (*p* < 0.001) and higher HD (*p* = 0.011) without significant difference in vs. (*p* = 0.660), compared to supratentorial PHE. In external test cohort, infratentorial ICH segmentation had lower mean Dice and vs. (both *p* < 0.001), and higher HD (*p* = 0.014), compared to supratentorial ICH. Similarly, infratentorial PHE segmentation had lower mean Dice and vs. (both *p* < 0.001), and higher HD (*p* = 0.005) compared to supratentorial PHE.

We also examined the potential association of lobar versus deep location of supratentorial hemorrhage on ICH and PHE segmentation results. In the Yale dataset, we found no significant difference in ICH segmentation Dice between lobar (0.94 ± 0.05) versus deep (0.92 ± 0.05) hemorrhage (*p* = 0.841), but slightly higher PHE segmentation Dice of lobar (0.71 ± 0.06) compared to deep (0.68 ± 0.09) hemorrhage (*p* = 0.002). In the Charité Hospital external testing cohort, we also found no significant difference in ICH segmentation Dice between lobar (0.83 ± 0.15) versus deep (0.85 ± 0.13) hemorrhage (*p* = 0.074) but slightly lower PHE segmentation Dice of lobar (0.62 ± 0.14) compared to deep (0.64 ± 0.12) hemorrhage (*p* = 0.028). Notably, the rate of lobar and deep hemorrhage was similar in ATACH-2, Yale, and Charité Hospital datasets (Supplementary Table S1).

We found no significant association between the CT scan parameters—specifically the slice thickness and axial plane voxel dimensions—with either ICH or PHE segmentation Dice in internal or external test cohorts. Specifically, the in-plane surface area size of voxels had no significant association with ICH (*p* = 0.471) or PHE (*p* = 0.230) segmentation Dice in Yale dataset, and ICH (*p* = 0.486) or PHE (*p* = 0.411) segmentation Dice in the Charité Hospital external testing cohort. The CT scan slice thickness also had no significant association with ICH (*p* = 0.655) or PHE (*p* = 0.062) segmentation Dice in the Yale dataset and ICH (*p* = 0.081) or PHE (*p* = 0.531) segmentation Dice in the Charité Hospital external testing cohort.

Examining the association between the time from onset to CT scan with segmentation Dice coefficient, we found no significant association between onset-to-scan time and the ICH (*p* = 0.714) or PHE (*p* = 0.193) segmentation Dice in the internal test cohort, nor in the external test cohort (*p* = 0.777 for ICH and *p* = 0.915 for PHE).

Finally, given that the age-related cumulative burden of cerebral small vessel disease and leukoaraiosis may hamper the accuracy of PHE segmentation, we examined the association between the patient’s age and the Dice coefficient of ICH and PHE segmentations. We found no significant association between patients’ age and ICH (*p* = 0.785) or PHE (*p* = 0.180) segmentation Dice in the internal test cohort, nor in the external unseen test cohort (*p* = 0.505 for ICH and *p* = 0.396 for PHE).

## 4. Discussion

We have developed an end-to-end automated pipeline for the segmentation of ICH and PHE in head CTs, which are the key imaging markers of primary and secondary brain injury in hemorrhagic stroke. In designing the deep learning model, we combined transformer and U-shaped CNN architectures and incorporated uncertainty metrics to ensure high accuracy and confidence in segmentation. By evaluating our model on large, multicentric datasets from institutions different from those that provided training scans, we validated model performance generalizability and achieved accuracies comparable to or exceeding prior studies. To facilitate inter-institutional collaborations, we packaged the final model along with the necessary libraries into a GUI application and publicly shared it on the NITRC data repository.

Hematoma and surrounding edema volumes as respective markers of primary and secondary brain injury after ICH can play a major role in treatment guidance and clinical trial enrollment. Almost all ICH clinical trials have used hematoma volume cutoff thresholds in their enrollment criteria [9,27,28,29,30,31]. Most recently, the Early Minimally Invasive Removal of Intracerebral Hemorrhage (ENRICH) Trial, which reported the benefits of hematoma evacuation, included patients with 30 to 80 mL supratentorial ICH in its enrollment criteria [32], highlighting the need for timely hematoma quantification to guide treatment in acute ICH.

Following primary injury from hemorrhage volume mass effects, inflammatory and cytotoxic responses to ICH hemoglobin breakdown byproducts can lead to secondary injury to surrounding parenchyma over in following days to weeks, presenting a potential window for therapeutic intervention. In addition to surgical interventions to reduce the hematoma’s mass effect, research into novel therapies targeting secondary brain injury in ICH is ongoing [6]. Clinical trials and case–control studies have shown reduced PHE growth following treatment with fingolimod [33], celecoxib [34], statin [35], hypertonic saline [36], and anti-adrenergic [37] therapies. PHE is also a recommended primary outcome for clinical trials evaluating hemostatic agents in ICH [38]. Accurate, reproducible, and efficient quantification of ICH and PHE volumes from CT scans can facilitate timely patient triage and early intervention in future clinical trials, especially since automated tools for medical image analysis and patient screening have been shown to enhance clinical trial enrollment rates [39]. Automated ICH and PHE quantification tools such as our GUI application can then facilitate timely treatment guidance and clinical trial enrollment in hemorrhagic stroke.

Since its inception in 2015 [40], the U-shaped network architecture (U-Net) has been the backbone of almost all deep learning segmentation models in medical imaging. U-Net applies a symmetric encoder–decoder structure with skip connections, effectively converging multiscale features in volumetric clinical scans. Many groups have applied U-Net-based architectures with different modifications for ICH segmentation, aiming to improve the segmentation accuracy. Table 2 summarizes the results of select prior studies of ICH and PHE segmentation.

Some groups have proposed U-Net models incorporating residual connections [41], DenseNet architecture [42], and capsule network [43] to benefit from structural characteristics of these networks. For example, Siddiquee et al. applied 2D SegResNet for 3D segmentation of ICH in head CTs [41]. SegResNet is a semantic segmentation network with residual connections, which helps avoid the vanishing gradient in deep networks by allowing information to skip layers and making it easier to train deeper models. Xu et al. [42] used Dense U-Net for the segmentation of ICH, extradural, and subdural hemorrhage in head CT. Inspired by DenseNet architecture, Dense U-Net connects each layer to every other layer in a feed-forward fashion, enhancing feature propagation and efficiency. Wang et al. [43] proposed a grouped capsule network named GroupCapsNet to segment ICH from non-contrast head CT scans. Compared to traditional CNNs, capsule networks can better preserve the spatial relationship information in 3D images and handle complex geometry [53]. Wang et al. [43] also modified the squashing function to accelerate the forward process in the training of the model without decreasing the performance.

Some authors modified the model framework to improve segmentation of smaller ICH lesions. For example, Gong et al. [44] applied the multi-scale learning-to-rank framework within U-Net structure to enhance the local feature discrimination of 3D patches containing ICH lesions. Their approach could specifically improve segmentation accuracy of tiny ICH (volume less than 1 mL).

To address the limitations of standard CNNs in capturing long-range dependencies, Peng et al. [45], incorporated attention mechanisms into their ICH segmentation models. They proposed AttFocusNet [45], with a focus structure from YOLO-V5 and attention gate mechanism for segmentation of ICH in head CTs. The focus structure integrates image information into the channel space for convolution, and the complete image features without pooling are fused with the features after convolutional pooling to preserve the integrity of overall features during encoding. Then, the attention gate will suppress redundant low-level features in output of the focus structure, capturing the most important features for the segmentation task. However, a limitation of their work was the segmentation of 2D ICH areas on individual CT slices separately, rather than 3D volumetric segmentation [45].

In 2017, transformers, using a self-attention mechanism, were introduced for natural language processing tasks [54] and were later adopted for computer vision tasks [55]. Recently, researchers have incorporated transformer blocks in segmentation models for medical images. Piao et al. [48] proposed TransHarDNet for ICH segmentation in head CTs, where they used HarDNet as the backbone of the encoder-decoder in a U-Net-shaped network (due to its lightweight architecture) and replaced convolution with a transformer block that connected the encoder and the decoder. Ma et al. [49] applied SwinUNET for PHE segmentation in a public dataset of head CTs (PHE-SICH-CT-IDS). The Swin UNEt TRansformers (Swin UNETR) [56] is a U-shaped network design that uses a Swin transformer as the encoder and a CNN-based decoder that is connected to the encoder via skip connections at different resolutions. In 2021, Isensee et al. introduced the nnU-NET model [57], a U-Net-based segmentation framework capable of automatically configuring itself for new segmentation tasks, including preprocessing, network architecture, training, and postprocessing. In the 2022 intracranial hemorrhage segmentation on non-contrast head CT (INSTANCE 2022) grand challenge held in conjunction with the International Conference on Medical Image Computing and Computer Assisted Intervention (MICCAI), nnU-NET based model achieved the best performance for ICH segmentation, outperforming U-Net, Attention U-Net and ResUNet [50]. Zhao et al. [51] also evaluated nnU-NET for fully automated segmentation of ICH, IVH, and PHE on non-contrast head CT. And, Kok et al. [52] proposed a 3D nnU-NET with Focal loss function for segmentation of ICH, PHE, and intraventricular hemorrhage (IVH) in head CTs. They applied that Focal loss function to address class imbalance and improve IVH segmentation when present. Notably, other groups have also proposed adaptive segmentation models for efficient organ segmentation as well as frameworks for solving nonlinear classification problems in medical datasets [58,59,60,61,62].

Our work addresses key knowledge gaps in existing reports on ICH and PHE segmentation. To date, most groups have focused on ICH segmentation, with only a limited number of reports on PHE segmentation models. Our study is one of the few to address both simultaneously. We are the first to apply a hybrid model combining transformers and CNNs for this task, building on recent reports of the advantages of such a dual structure in skin lesion segmentation [19]. Indeed, the hybrid model also outperformed each network model in our study (Table 1). Another important and previously unexplored aspect of our work was the incorporation of prediction uncertainty metrics, allowing us to identify subjects where the model had low confidence in ICH and PHE segmentation results. Recognizing that many research groups may lack the expertise to locally deploy segmentation codes, we developed and shared a user-friendly GUI application for local implementation, making our model more accessible and addressing an unmet need for clinical researchers. Finally, we validated our approach on the largest external cohort ever used for ICH and PHE segmentation (Table 2), providing a realistic and reliable assessment of its performance.

Since their inception in 2017, transformers have achieved remarkable success in natural language processing [54]. Inspired by this success, researchers have introduced Transformers into computer vision, leading to development of models such as Vision Transformer [55], and Swin Transformer [18]. More recently, Transformers have been adopted for medical image segmentation, including Swin-Unet [63], which is a purely Transformer-based structure for both the encoder and decoder in 2D image segmentation and Swin Unet 3D for volumetric medical image segmentation [64]. Most recently, hybrid models combining transformers and CNN have shown superior performance in medical image segmentation tasks over each model architecture [19]. Motivated by these results we aimed to apply a hybrid transformer-CNN model structure for segmentation of ICH and PHE in head CTs and compare its performance with benchmark models. The self-attention mechanisms in transformers are particularly efficient at capturing global and long-range dependencies within input images. This is particularly valuable where important features are spatially distant. On the other hand, CNNs are highly efficient in learning local features such as edges, textures, and small patterns, which are important for segmenting fine structures in medical scans. The local feature extraction ability and hierarchical feature learning nature of CNNs can provide detailed spatial information that complements the global context captured by transformers in segmentation tasks.

We integrated SwinUNETR [56], originally proposed for 3D brain tumor segmentation, into nnU-Net [57], a U-shaped segmentation model that can automatically configure itself, including preprocessing, network architecture, and postprocessing [57]. In addition to network architecture, image pre-processing plays an important role in the performance of medical image analysis models. The nnU-Netv2 offers a flexible and modular architecture that can be tailored to different datasets and segmentation tasks, as it inherently provides pre-processing pipeline, data augmentation, and various 3D neural network architectures. We have shown that the dual Swin-nnUNET model achieves better segmentation performance metrics compared to SwinUNETR. The dual Swin-nnUNET also achieved lower HD, but similar Dice and VS metrics, compared to nnU-Net.

Our proposed model leverages the collaborative training of different deep learning models and uses multiple output resolutions for loss functions to enhance the accuracy and robustness of segmentations. Different resolutions capture information at different scales. While feature maps of original resolution can capture fine-grained details, low-resolution feature maps capture more global information. By using multiple output resolutions, the model can capture detailed and global features of the input image.

We also incorporated measures of prediction uncertainty into our model. Beyond accuracy, prediction uncertainty is a critical metric when deploying deep learning models in healthcare. While segmentation accuracy is a key indicator of model performance, it is equally important to identify cases with high prediction uncertainty to enhance the reliability of the results. Estimating uncertainty helps detect ambiguities and potential errors in predictions, thereby increasing confidence in the model’s outputs. Measures of prediction uncertainty have been notably absent in previous reports on ICH and/or PHE segmentation. We address this issue by implementing a test-time augmentation framework [65] to quantify ICH and PHE segmentation prediction uncertainty.

We applied test-time augmentation to identify uncertainty in segmentation results [23,24,65,66,67]. Test-time augmentation allows the segmentation model to evaluate images from multiple perspectives, effectively increasing data diversity during inference. Techniques such as rotation or zooming can make small objects or boundaries more prominent in certain augmented versions. When training data are limited or the model is not perfectly trained, test-time augmentation helps reduce errors by leveraging these augmented perspectives during inference [23,24,65,66,67]. Additionally, combining predictions from augmented images acts as a form of aggregation, improving accuracy without requiring multiple separate models. Compared to other methods for uncertainty quantification, which often require retraining or modifications to the model, test-time augmentation is computationally inexpensive as it involves no changes to the model architecture or additional training. However, test-time augmentation does increase inference time due to the need to process multiple versions of each input.

High uncertainty scores indicate the need for visual review and manual editing of segmentations. After removing scans with uncertainty, the segmentation performance metrics further improved among the remaining subjects with high-confidence segmentations. We have demonstrated that subjects with higher uncertainty in segmentation predictions had lower accuracy, as reflected by lower Dice and VS and higher HD (see Supplementary Table S3). Uncertainty filtering—by identifying and excluding instances where the model’s confidence is low—typically eliminates ambiguous predictions or noisy data. By focusing on high-confidence predictions, deep learning models operate within a domain where they are most reliable, thereby reducing the overall error rate. This strategy can increase the overall accuracy in remaining subjects and improve the model’s robustness and reliability in clinical predictions, where precision is critical.

We also demonstrated the feasibility of using GUI applications for external unseen testing and inter-institutional collaboration, overcoming data-transfer obstacles and lack of technical expertise for efficient model implementation in other centers. Moreover, the large size of the training and validation cohorts, along with the completely isolated nature of both internal and external unseen testing cohorts, suggests that our results are generalizable. Finally, it is important to note that accurate quantification of ICH and PHE volumes is often more clinically relevant than precise segmentation of lesions. Therefore, the high VS of our model’s segmentations in validation cohorts is particularly valuable for translation to clinical settings and guiding treatment decisions.

The model segmentation performance was slightly hampered in the external unseen dataset (Table 1 and Table 2), which may be in part due to inherent differences between that cohort with ATACH-2 and Yale datasets. This contrasts with independent validation cohorts used by other groups (Table 2) [42,43,45,48,51,52], where the dataset is typically split for training and validation, leading to more homogeneous characteristics of train and test cohorts and, thus, improving the overall model performance in their test cohorts.

We explored factors that may affect the accuracy of lesion segmentations and found that larger IVH volumes were associated with lower Dice coefficients for ICH and PHE segmentation in the external test cohort, but not in the internal test set. This likely reflects the intrinsic challenges of separating intraparenchymal from intraventricular hemorrhage. We also found that the model’s accuracy and certainty were lower for infratentorial ICH and PHE lesions. Overall, 10 to 15% of all ICH cases are infratentorial [5]. However, under-representation of infratentorial ICH in the training dataset (Supplementary Table S1) may have contributed to the model’s reduced performance in segmenting infratentorial ICH and PHE lesions. Notably, there was no significant difference in ICH segmentation Dice between lobar versus deep hemorrhage.

Although the ICH and PHE volumes in the training dataset were smaller than those in the external test cohort, we found no association between ICH volume and HD in either the internal or external test cohorts; and larger PHE volumes were even associated with lower HD (better segmentation) in the external test cohort. On the other hand, uncertain segmentations had smaller ICH and PHE volumes. It should be noted that during the training process, we applied data augmentation strategies such as scaling, rotation, and translation. These techniques helped CNN recognize hematoma and edema lesions across various orientations and sizes. CNNs are designed to learn hierarchical feature representations from the data, identifying key structures such as edges, textures, and shapes that characterize the hematoma and surrounding edema. Thus, the smaller ICH volumes in the training dataset did not preclude accurate segmentation of larger hematomas in the test cohort.

Manual delineation of lesions on medical images, despite its imperfections, remains the gold standard for training and testing automated segmentation models. Although both research teams applied similar manual segmentation processes, slight differences are inevitable and may have contributed to lower segmentation accuracy in external unseen cohorts. It is noteworthy that there were good to excellent inter- and intra-rater agreements in the segmentation of ICH and PHE lesions by both research teams. The Yale team reported mean ICCs ranging from 0.92 to 0.94 for ICH segmentation and 0.93 to 0.94 for PHE segmentation [10,11,12]; the Charité Hospital team reported ICCs ranging from 0.97 to 0.99 for ICH and 0.89 to 0.98 for PHE segmentation [14,15,16].

There are other limitations to our study. Since the majority of head CTs in this study were obtained within the first 36 h after ICH onset and the imaging characteristics of ICH and PHE evolve through the subacute and chronic stages, our model’s performance is likely suboptimal in extended time windows. However, it is noteworthy that we found no association between the time-to-scan gap and the accuracy of segmentations in our test cohorts. In addition, while PHE volume typically peaks between 3 days and 3 weeks after hemorrhage [6], the initial phase of rapid PHE growth in ICH patients is reported to occur within the first 24 h post-onset [68,69,70]. Thus, many interventions aimed at reducing secondary brain injury and PHE growth are targeted during this acute phase, highlighting the clinical relevance of PHE quantification during this time window. Another limitation of this study was the lack of testing on public datasets. However, such datasets typically lack detailed information about the patient cohort, the time from onset to scan, and the specifics of ground truth segmentation, which are crucial for comparison of different models’ performance. It also remains to be established if the model can be extended to other forms of cerebral hemorrhage such as traumatic brain injury, hemorrhagic metastasis, or hemorrhagic transformation of ischemic infarct. Finally, manual delineation of PHE from pre-existing leukoaraiosis imposes a technical limitation. MRI scans were not available to assist with the delineation of PHE and its distinction from leukoaraiosis. Our teams relied on the distribution of hypodense PHE, which surrounds hyperdense ICH but appears slightly denser than the normal brain tissue versus leukoaraiosis, which typically appears as diffuse or patchy hypodensity. Notably, we found no association between PHE segmentation accuracy and patients’ age, as a surrogate for cumulative burden of cerebral small vessel disease and leukoaraiosis. After applying nnU-Net to segment ICH and PHE from CT scans, the next steps will emphasize predicting clinical outcomes. These include quantifying ICH and PHE volumes to monitor disease progression or treatment response and analyzing the spatial relationship between ICH, PHE, and adjacent brain structures to gain deeper clinical insights.

## 5. Conclusions

We developed and validated an end-to-end pipeline for automated segmentation of hematoma and edema in acute spontaneous ICH on head CTs and incorporated the model in an easy-to-use GUI application for external unseen testing by other collaborators. The application is publicly shared on NITRC data repository website. The integration of the SwinUNETR transformer and versatile nnU-NETv2 with metrics of uncertainty improved model accuracy and confidence in segmentation results. The segmentation accuracy was hampered by larger IVH volume and infratentorial location of hemorrhage. Segmentation uncertainty was associated with infratentorial location and smaller ICH and PHE volumes. With a high VS index in both internal and external unseen testing, our model has practical capability to facilitate prognostication and guide treatment decisions in acute hemorrhagic stroke patients based on admission head CT scans.

## Figures and Tables

**Table 1:** Summary of models’ segmentation performance for (A) ICH and (B) PHE.

Method	Testing Data	A. ICH Segmentation					
		Dice		VS		HD (mm)	
		Mean	Median	Mean	Median	Mean	Median
SegResNet	Cross-validation	0.85 ± 0.07	0.87 (0.81–0.91)	0.91 ± 0.41	0.94 (0.90–0.95)	5.2 ± 20.1	2.2 (1.2–2.8)
	Internal testing	0.83 ± 0.17	0.87 (0.80–0.91)	0.88 ± 0.25	0.95 (0.89–0.96)	6.2 ± 13.1	1.9 (0.9–5.1)
SwinUNETR	Cross-validation	0.86 ± 0.17	0.9 (0.84–0.92)	0.92 ± 0.40	0.95 (0.91–0.96)	4.9 ± 18.2	1.9 (1.5–2.4)
	Internal testing	0.84 ± 0.18	0.89 (0.84–0.92)	0.89 ± 0.17	0.96 (0.90–0.97)	5.5 ± 12.1	1.6 (0.1–5.0)
	External unseen testing	0.73 ± 0.23	0.81 (0.68–0.87)	0.78 ± 0.23	0.86 (0.74–0.92)	8.7 ± 10.8	5.0 (2.9–8.8)
nnUNET	Cross-validation	0.88 ± 0.07	0.90 (0.85–0.92)	0.93 ± 0.35	0.95 (0.92–0.97)	3.4 ± 15.2	1.7 (1.5–2.4)
	Internal testing	0.90 ± 0.08	0.93 (0.90–0.95)	0.94 ± 0.10	0.97 (0.94–0.98)	3.5 ± 11.1	1.0 (0.5–2.4)
	External unseen testing	0.80 ± 0.18	0.86 (0.79–0.90)	0.86 ± 0.16	0.91 (0.85–0.95)	7.3 ± 12.1	4.0 (2.1–5.8)
SwinUNETR + nnU-NET	Cross-validation	0.89 ± 0.07	0.91 (0.87–0.93)	0.93 ± 0.20	0.96 (0.93–0.98)	2.9 ± 11.7	1.2 (0.7–2.2)
	Internal testing	0.90 ± 0.11	0.93 (0.90–0.95)	0.94 ± 0.10	0.97 (0.94–0.98)	2.3 ± 5.9	0.9 (0.5–1.9)
	External unseen testing	0.82 ± 0.17	0.86 (0.80–0.90)	0.86 ± 0.16	0.91 (0.85–0.95)	6.0 ± 9.2	4.0 (2.1–5.5)
Final model *	Internal testing	0.91 ± 0.08	0.93 (0.90–0.95)	0.95 ± 0.08	0.97 (0.95–0.98)	2.0 ± 5.1	0.9 (0.5–1.9)
	External unseen testing	0.83 ± 0.13	0.87 (0.81–0.91)	0.88 ± 0.12	0.91 (0.85–0.95)	5.8 ± 8.8	4.0 (2.1–5.4)
**Method**	**Testing** **data**	**B. PHE segmentation**					
		**Dice**		**VS**		**HD (mm)**	
		**Mean**	**Median**	**Mean**	**Median**	**Mean**	**Median**
SegResNet	Cross-validation	0.69 ± 0.11	0.71 (0.69–0.75)	0.87 ± 0.75	0.88 (0.84–0.91)	8.1 ± 17.1	5.5 (4.1–6.0)
	Internal testing	0.60 ± 0.71	0.65 (0.60–0.69)	0.79 ± 0.22	0.84 (0.74–0.90)	7.1 ± 10.1	5.9 (3.1–6.3)
SwinUNETR	Cross-validation	0.70 ± 0.28	0.71 (0.69–0.75)	0.87 ± 0.55	0.89 (0.84–0.92)	7.9 ± 18.3	5.3 (4.1–6.1)
	Internal testing	0.61 ± 0.16	0.65 (0.58–0.70)	0.80 ± 0.19	0.86 (0.75–0.93)	6.9 ± 8.8	5.0 (3.3–6.0)
	External unseen testing	0.51 ± 0.18	0.56 (0.44–0.64)	0.74 ± 0.24	0.82 (0.66–0.91)	9.3 ± 10.2	5.7 (4.1–10.7)
nnU-NET	Cross-validation	0.74 ± 0.28	0.75 (0.70–0.79)	0.88 ± 0.35	0.90 (0.85–0.93)	6.0 ± 15.3	3.5 (2.4–4.5)
	Internal testing	0.66 ± 0.10	0.68 (0.62–0.78)	0.80 ± 0.13	0.82 (0.73–0.9)	7.3 ± 13.8	4.6 (3.4–5.3)
	External unseen testing	0.60 ± 0.15	0.65 (0.56–0.70)	0.80 ± 0.17	0.84 (0.73–0.92)	9.1 ± 14.3	5.0 (3.8–7.5)
SwinUNETR + nnU-NET	Cross-validation	0.75 ± 0.09	0.76 (0.71–0.8)	0.89 ± 0.23	0.91 (0.86–0.94)	5.1 ± 15.3	2.8 (2.0–4.2)
	Internal testing	0.68 ± 0.10	0.70 (0.64–0.75)	0.85 ± 0.10	0.87 (0.80–0.93)	5.2 ± 6.7	4.5 (3.0–5.2)
	External unseen testing	0.60 ± 0.15	0.65 (0.56–0.70)	0.81 ± 0.17	0.85 (0.75–0.93)	6.8 ± 7.7	4.9 (3.6–7.3)
Final model *	Internal testing	0.69 ± 0.08	0.70 (0.64–0.75)	0.86 ± 0.10	0.87 (0.80–0.93)	5.0 ± 6.2	4.5 (3.0–5.1)
	External unseen testing	0.62 ± 0.13	0.65 (0.56–0.70)	0.83 ± 0.14	0.86 (0.77–0.93)	6.7 ± 6.9	4.9 (3.6–7.2)

* A total of 7 (out of 396) segmentations in the internal testing cohort and 24 (out of 943) in the external unseen testing cohort were excluded for uncertainty. The cross-validation cohort from the ATACH-2 trial included n = 1782 CTs.

**Table 2:** Summary of prior studies on automated segmentation of ICH and PHE.

Model	Architecture	Testing Data	ICH		PHE	
			Dice	HD	Dice	HD
2D SegResNet [41]	UNET + Residual connections to avoid vanishing gradient	30 from INTANCE Challenge (validation)	0.72			
Dense U-Net [42]	U-Net + Dense connections for better feature reuse	211 (independent)	0.90			
		86 (external)	0.86			
GroupCapsNet [43]	Capsule-Net to preserve spatial relationship	210 (5-fold cross-validation)	0.88			
U-Net with Learning-to-rank module [44]	Multi-scale learning-to-rank framework within U-Net to improve tiny ICH segmentation	267 (validation)	0.73			
AttFocusNet [45]	Focus structure from YOLO-V5 to capture all features + Attention gate to suppress low-level redundant features	207 (independent)	0.88	5.33		
		50 from CQ500 [46] and RSNA 2019 [47] dataset (external)	0.91	4.22		
TransHarDNet [48]	HarDNet as the backbone of U-shaped network + transformer blocks instead of convolution	1200 slices (independent)	0.71			
Swin-Unet [49]	Swin transformer encoder and a CNN-based decoder	703 slices (independent)			0.35	47.8
nnU-NET [50]	nnU-Net outperformed U-Net, Attention U-Net and ResUNet	30 from INTANCE Challenge (validation)	0.79	21.6		
nnU-NET [51]	3D nnU-NET	80 (independent)	0.92		0.71	
nnU-NET + Focal loss function [52]	nnU-NET + focal loss function to address class imbalance and improve IVH segmentation	174 (independent)	0.90		0.61	
Our Swin-nnUnet with uncertainty detection	Swin-Unet + nnU-Net (Figure 2) and test-time augmentation for uncertainty detection	396 Internal independent tests	0.91	2.0	0.69	5.0
		943 External independent unseen datasets	0.83	5.8	0.62	6.7

## Data Availability

Data are available upon request and on approval from the respective register holders. Model architecture is available at: https://github.com/anhtrnyaleedu/ICH_PHE_segmentation/blob/main/model_architecture.png (accessed on 20 May 2024).

## Supplementary Materials

The following supporting information can be downloaded at: https://www.mdpi.com/article/10.3390/bioengineering11121274/s1, Multiple loss function for multiple output segmentation; Evaluation of model performance; Supplementary Table S1. The demographic and clinical characteristics of patients in training/cross-validation versus test cohorts; Supplementary Table S2. Deep learning model segmentation performance tabulated for supra- and infratentorial hemorrhage; Supplementary Table S3. Comparison of segmentation performance between subject with and without uncertainty in prediction based on test-time augmentation and uncertainty quantification; Example of Dice diagram, loss diagram, time during training 5-folds cross-validation.
