# Supplementary material for: A Hybrid Transformer-Convolutional Neural Network for Segmentation of Intracerebral Hemorrhage and Perihematomal Edema on Non-Contrast Head Computed Tomography (CT) with Uncertainty Quantification to Improve Confidence
Source: Bioengineering (Basel). 2024 Dec 15;11(12):1274. doi: 10.3390/bioengineering11121274 (PMC11672977; doi:10.3390/bioengineering11121274)
Supplement: Supplementary file 1 [file bioengineering-11-01274-s001.zip › bioengineering-3339790-supplementary.pdf]

1 Multiple loss function for multiple output segmentation

- The Cross-Entropy Loss: A popular loss function for (pixel-wise) classification problems is the Log-loss, also known as Cross-Entropy loss, which is defined as:

$$L_{CE} = -\sum_{c=1}^{C} w_c log \frac{\exp(x_{n,c})}{\sum_{i=1}^{C} \exp(x_{n,i})} y_{n,c} \quad (1)$$

where x is the input, y is the target, w is the weight, and C is the number of classes.

- Dice Similarity Coefficient (DSC) measures the volumetric overlap between segmentation results and ground truth. Dice is computed where A is the set of foreground voxels in the ground truth and B is the corresponding set of foreground voxels in the segmentation result.

$$L_{Dice} = 1 - \frac{2(A \cap B)}{|A| + |B|} \quad (2)$$

2 Evaluation of model performance

Dice Coefficient measures the volumetric overlap between segmentation results and the ground truth. Dice is computed where A is the set of foreground voxels in the ground truth and B is the corresponding set of foreground voxels in the segmentation result.

$$Dice = \frac{2(A \cap B)}{|A| + |B|} \quad (3)$$

Hausdorff distance (HD) which measures surface distance is the maximum distance of a set to the nearest point in the other set defined as

$$d_H(X,Y) = \max\{d_{XY}, d_{YX}\} = \max\left\{\max_{x\in X} \min_{y\in Y} d(x,y), \max_{y\in Y} \min_{x\in X} d(x,y),\right\} \quad (4)$$

Volume Similarity measures and compares the absolute volume of the segmented result and ground truth, defined as

$$VS = 1 - \frac{|v1 - v2|}{v1 + v2} \quad (5)$$

3 **Supplementary Table 1. The demographic and clinical characteristics of patients in training/cross-validation versus test cohorts**

	**Training ATACH (893 patients)**	**Independent test Yale (202 patients)**	**Independent test Charité (943 CTs)**	**p-value**
Male	543 (60.8%)	103 (51.0%)	530 (56.4%)	1.000
Age* [years]	62.2 ± 13.1	70.6 ± 14.0	69.4 ± 14.2	<0.001
Systolic blood pressure* [mmHg]	171.6 ± 25.0	171.3 ± 38.1	136.3 ± 75.9	<0.001
History of hypertension	494 (80.9%)	172 (85.1%)	740 (78.5%)	0.317
History of diabetes mellitus	146 (23.9%)	52	150	0.971
NIHSS at baseline<br>0-4<br>5-9<br>10-14<br>15-19<br>20-25<br>>25<br>Unknown	101 (16.5 %)<br>157 (25.7 %)<br>157 (25.7 %)<br>108 (17.7 %)<br>71 (11.6 %)<br>13 (3.1 %)<br>2 (0.4 %)	75 (37.12%)<br>36 (17.82%)<br>35 (17.32%)<br>35 (17.32%)<br>19 (9.4%)<br>2 (1.02%)	306 (32,48%)<br>145 (15,39%)<br>181 (19,21%)<br>82 (8,70 %)<br>76 (8,06%)<br>16 (1,70%)<br>136 (14,43%)	<0.001
Onset to ct scan time (hours)	13.4 ± 4.0	9.8 ± 7.6	15.0 ± 66.7	<0.001
Supra-tentorial ICH (%)	892 (99.9%)	177 (87.6%)	787 (83.5%)	<0.001
Lobar (%)	106 (11.9%)	67 (34.4%)	424 (44.8%)	0.512
IVH (%)	246 (27.5%)	78 (38.6%)	310 (32.8%)	<0.001
IVH volume [mL]	2.4 ± 6.6	3.4 ± 8.6	15.5 ± 22.7	<0.001
ICH volume* [mL]	19.4 ± 40.4	15.717 ± 16.7	30.1 ± 32.6	<0.001
PHE volume* [mL]	19.4 ± 40.4	16.1 ± 14.4	29.4±29.8	<0.001
CT – mean ± SD<br>In-plane pixel spacing [mm]<br><br>Slice thickness [mm]<br>Min axial image matrix [n x n]<br>Max axial matrix [n x n]<br>Number of slices	 [0.47±0.08 × 0.47±0.08]<br>5.29 ± 1.81<br>[512 × 512]<br>[512 ×734]<br>30.9 ± 17.6	 [0.46±0.03 × 0.46±0.03]<br>4.93 ± 0.60<br>[472 × 472]<br>[512 ×715]<br>30.9 ± 17.6	 [0.46±0.04 × 0.46±0.04]<br>4.54 ± 0.66<br>[434 × 434]<br>[512 × 671]<br>30.26 ± 7.8	

*Using three-sample one way ANOVA test; others using the chi-square test

Supplementary Table 2. Deep learning model segmentation performance tabulated for supra- and infra-tentorial hemorrhage.

Method	Testing data	a. ICH segmentation					
		Dice		HD (mm)		VS	
		Mean	Median	Mean	Median	Mean	Median
**SegResNet**	Cross-validation	0.85±0.07	0.87 (0.81–0.91)	5.2±20.1	2.2 (1.2–2.8)	0.90±0.33	0.94 (0.90–0.97)
	Internal testing (supratentorial)	0.84±0.71	0.84 (0.80–0.90)	5.2±11.1	4.9 (1.9–6.1)	0.88±0.61	0.92 (0.87–0.94)
	Internal testing (infratentorial)	0.66±0.71	0.85 (0.79–0.88)	17.9±30.3	7.16 (1.9–5.1)	0.84±0.75	0.88 (0.84–0.92)
**SwinUNETR**	Cross-validation	0.86±0.17	0.9 (0.84–0.92)	4.9±18.2	1.9 (1.5–2.4)	0.91±0.07	0.94 (0.90–0.97)
	Internal testing (supratentorial)	0.85±0.15	0.90 (0.84–0.92)	4.5±8.6	1.5 (0.9–3.6)	0.90±0.14	0.96 (0.91–0.98)
	Internal testing (infratentorial)	0.67±0.33	0.84 (0.58–0.89)	15.6±28.4	9.3 (5.5–12.2)	0.75±0.32	0.90 (0.69–0.95)
	External testing (supratentorial)	0.76±0.19	0.83 (0.73–0.88)	8.1±10.3	5.0 (2.8–8.1)	0.81±0.19	0.87 (0.78–0.92)
	External testing (infratentorial)	0.57±0.30	0.70 (0.38–0.81)	11.8±17.2	5.2 (4.0–12.9)	0.64±0.30	0.74 (0.46–0.87)
**SwinUNETR + nnUNet & excluding those with uncertainty**	Cross-validation	0.89±0.07	0.91 (0.87–0.93)	2.9±11.8	1.2 (0.7–2.2)	0.93±0.17	0.94 (0.89–0.97)
	Internal testing (supratentorial)	0.91±0.07	0.93 (0.90–0.95)	1.9±5.2	0.9 (0.9–1.9)	0.96±0.07	0.97 (0.94–0.99)
	Internal testing (infratentorial)	0.85±0.17	0.90 (0.82–0.93)	3.1±4.3	1.2 (0.5–3.4)	0.90±0.17	0.95 (0.89–0.97)
	External testing (supratentorial)	0.85±0.11	0.88 (0.82–0.91)	5.2±8.0	3.7 (2.0–5.0)	0.89±0.11	0.91 (0.86–0.96)
	External testing (infratentorial)	0.77±0.15	0.82 (0.73–0.86)	7.1±10.1	4.4 (2.7–8.2)	0.81±0.18	0.87 (0.77–0.92)

Method	Testing data	b. PHE segmentation					
		Dice		HD (mm)		VS	
		Mean	Median	Mean	Median	Mean	Median
**SegResNet**	Cross-validation	0.69±0.11	0.7 (0.7–0.8)	8.1±17.1	5.5 (4.1–6.0)	0.86±0.23	0.87 (0.81–0.94)
	Internal testing (supratentorial)	0.61±0.71	0.65 (0.57–0.70)	7.1±19.1	8.9 (6.1–15.3)	0.82±0.21	0.86 (0.75–0.92)
	Internal testing (infratentorial)	0.47±0.71	0.54 (0.45–0.68)	11.1±22.3	7.9 (3.3–10.1)	0.71±0.35	0.80 (0.70–0.94)
**SwinUNETR**	Cross-validation	0.70±0.28	0.71 (0.69–0.80)	7.9±18.3	5.3 (4.1–6.2)	0.87±0.10	0.88 (0.81–0.94)
	Internal testing (supratentorial)	0.62±0.14	0.66 (0.58–0.70)	6.5±6.8	5.01 (3.4–5.8)	0.82±0.17	0.86 (0.76–0.93)
	Internal testing (infratentorial)	0.48±0.23	0.55 (0.44–0.63)	10.1±19.9	5.02 (3.9–8.4)	0.71±0.32	0.81 (0.69–0.95)
	External testing (supratentorial)	0.55±0.16	0.59 (4.9–6.5)	9.0±10.0	5.5 (4.1–10.0)	0.78±0.20	0.84 (0.70–0.92)
	External testing (infratentorial)	0.34±0.21	0.39 (0.17–0.52)	11.1±10.6	8.2 (4.9–14.0)	0.57±0.33	0.68 (0.27–0.85)
**SwinUNETR + nnUNet & excluding those with uncertainty**	Cross-validation	0.75±0.09	0.76 (0.71–0.8)	5.1±14.6	2.8 (2.0–4.2)	0.92±0.16	0.93 (0.86–0.94)
	Internal testing (supratentorial)	0.69±0.08	0.71 (0.64–0.75)	5.0±6.4	4.3 (3.0–5.1)	0.86±0.10	0.87 (0.80–0.93)
	Internal testing (infratentorial)	0.67±0.07	0.65 (0.60–0.74)	5.45±3.73	5.0 (4.1–5.9)	0.86±0.09	0.87 (0.81–0.93)
	External testing (supratentorial)	0.64±0.11	0.66 (0.59–0.71)	4.7±4.1	4.8 (3.6–6.3)	0.83±0.14	0.86 (0.77–0.93)
	External testing (infratentorial)	0.54±0.11	0.57 (0.46–0.64)	7.4±5.8	5.7 (4.6–9.8)	0.79±0.20	0.85 (0.68–0.91)

Supplementary Table 3. Comparison of segmentation performance between subject with and without uncertainty in prediction based on test-time augmentation and uncertainty quantification.

**Internal test segmentation (n=396)**		**With uncertainty (n=7)**	**Without uncertainty (n=389)**	**P value**
**ICH**	Dice	0.47±0.40	0.91±0.09	<0.001
	VS	0.64±0.26	0.95±0.09	<0.001
	HD (mm)	15.7±19.3	2.0±5.1	<0.001
**PHE**	Dice	0.31±0.27	0.69±0.09	<0.001
	VS	0.81±0.13	0.86±0.10	0.249
	HD (mm)	17.8±17.2	5.0±6.2	<0.001
**Internal test segmentation (n=943)**		**With uncertainty (n=24)**	**Without uncertainty (n=919)**	
**ICH**	Dice	0.27±0.35	0.83±0.13	<0.001
	VS	0.31±0.38	0.88±0.13	<0.001
	HD (mm)	12.3±19.4	5.8±8.8	<0.001
**PHE**	Dice	0.19±0.22	0.62±0.13	<0.001
	VS	0.35±0.37	0.83±0.15	<0.001
	HD (mm)	14.4±21.9	6.7±6.9	<0.001

6 Example of Dice diagram, loss diagram, time during training during training 5-folds cross-validation

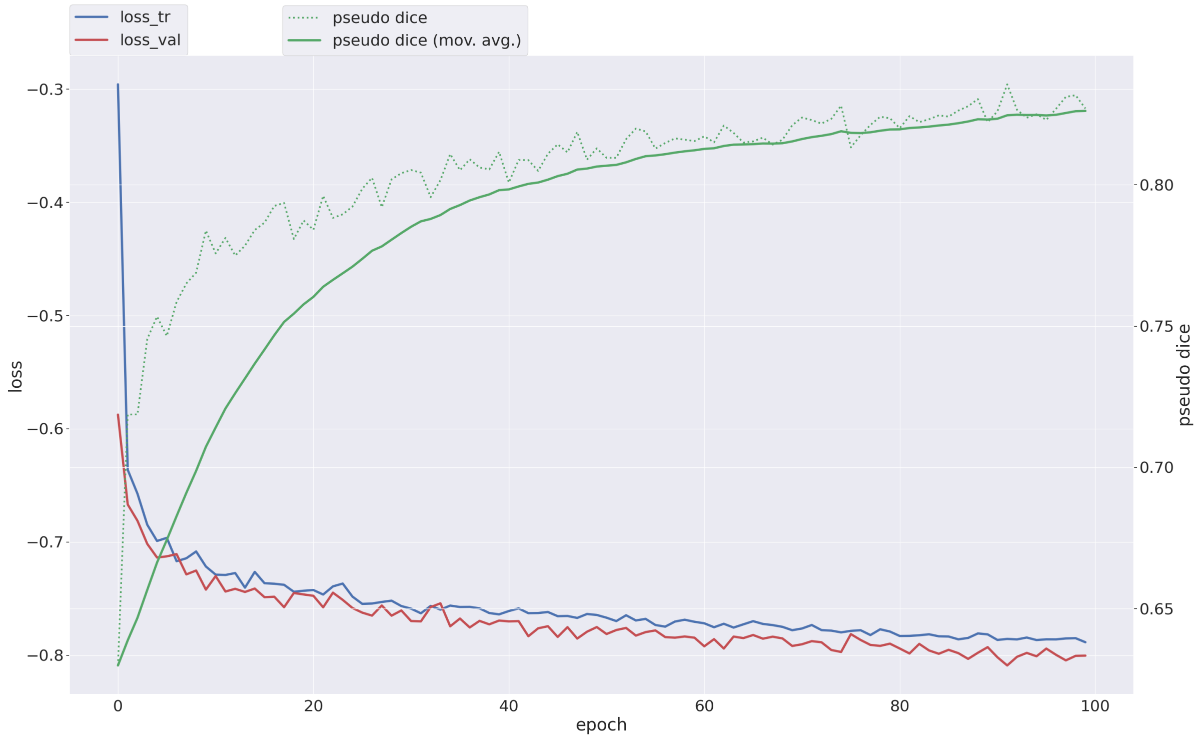

7 Model Architecture

https://github.com/anhtrnyaleedu/ICH_PHE_segmentation/blob/main/model_architecture.png
